# The impact on physical performance, pain and psychological wellbeing of chronic low back pain patients during 12-weeks of equine- facilitated therapy intervention

**DOI:** 10.3389/fvets.2023.1085768

**Published:** 2023-03-14

**Authors:** Sanna Mattila-Rautiainen, Mika Venojärvi, Heta Rautiainen, Alice Keski-Valkama

**Affiliations:** ^1^Sports and Exercise Medicine, University of Eastern Finland, Kuopio, Finland; ^2^Swedish University of Agricultural Sciences, Upsala, Sweden; ^3^Vanha Vaasa Hospital, Vaasa, Finland

**Keywords:** animal-assisted therapy, human-animal bonding, wellbeing, performance, mental health

## Abstract

Equine - Facilitated Therapy (EFT), an equine environment, and horses themselves can meet many physical and mental health needs beyond diagnostic categories. The horse's ability to produce a walk-like movement and the participant's ability to connect to non-judgemental living creatures, both of which can benefit participation and construct a positive self-image for chronic pain patients. The objective of this study is to evaluate the impact of EFT on perceived physical performance, level of pain, pain acceptance, depression and anxiety, and quality of life within a 12-week intervention for chronic low back pain (LBP) patients. Twenty-two LBP patients received EFT led by physical therapists as part of the public health services. A mixed method design combining quantitative and qualitative methods was employed to detect the outcome of the intervention. The data were collected *via* questionnaires, interviews, and patient data repositories. An interview was voluntary for participants and included questions of one's health, visits to the pain clinic during 6 months and an open-ended question about the intervention. The coding of the data was completed independently by two persons using thematizing. The welfare of the attending horses was taken into consideration in basic training and for the research setting. Statistical analysis and paired *t*-tests detected the changes during a 12-week intervention. The results suggest a significant increase in Canadian Occupational Performance Measure (COPM) levels of satisfaction with self-selected performances. The Raitasalo's version of Beck's Depression Inventory (RBDI) level of anxiety and Chronic Pain Acceptance Questionnaire (CPAQ) did not change, whereas a decline in the amount of perceived RBDI depression was found combined with increased levels of SF-36 Mental Change Scores and COPM satisfaction with performance. Only two of the 22 participants returned with reoccurring symptoms after 6 months to the pain clinic. The participant interviews revealed three important domains of experience during coding: physical-, psychological-, and social that link to the research question and suggest impact for the recovery from the human-animal interaction.

## 1. Introduction

Pain-related fear may lead to avoidance of spinal motion for chronic low back pain (LBP) patients (1) and limit daily physical activities like social contact, self-care and communication (2). Avoidance of performance, high levels of kinesiophobia and irrational fear of movement cause further physical disability with elevated levels of pain (3) as fear motivates defensive responses such as escape from an external stimulus (4). Physical exercise is found to be the most effective treatment for the management of spinal pain, offering small to moderate short-lasting treatment effects (5). Additionally, medication is widely used to provide significant pain relief, and patients with prolonged pain tend to take multiple medicines (6). Management of life and pain (7) is important not only at the individual level but also economically at the society level, as the prevalence of low back pain, together with mental health and social problems is high (8). Psychological wellbeing is the connection between physical and psychological functioning (9), as cognitions and emotions play a significant role in the perception of chronic pain (10) and body awareness (11). Emotions are produced by processes which include emotional awareness, labeling, expression, processing, and integration influencing mental, behavioral and physical health (12). Sophisticated models recognize the brain as an organ where pain is modulated. It develops a basis of psychosocial and biological processes allowing individuals to experience and report somatic pain (13).

The process of learning cognitive, emotional, and environmental influences and experiences are key elements for a change (14), suggesting that chronic LBP is a multidimensional biopsychosocial problem including various factors, such as negative pain cognitions, pain-related fear and emotional distress, avoidance and protective behaviors in movement, and sleep problems (15). Physical exercise therapy is the most recommended treatment for chronic LBP if the patients are matched with the appropriate exercise and safety for recovery (16, 17). The recommendation for patients with chronic LBP who do not benefit from primary care treatment should be referred to interdisciplinary biopsychosocial pain rehabilitation in secondary care settings (15).

Therapy conducted with an equine is not usually the first recommendation of physical therapy for an LBP patient (18) despite history being rich with horses' curative effects (19). However, orthopedic hippotherapy has already been used as a conservative treatment for segmental instabilities in the lumbar section (20) as sitting on a moving horse has specificity for the exercise producing 100–120 walk-like movements per minute for the person sitting astride (21). Equine-facilitated therapy is more known as a treatment for balance (22), kinematics of walking (2), symmetry (23) and gross motor function (24) for neurological disorders. The treatment has been recognized for its psychological effects like the quality of life (25), a reduction in the amount of pain in LBP patients (26) also utilizing mechanical horse (27).

Companion animals (28) and connectedness to nature (29) have been found to affect human psychological, behavioral, physiological, and social wellbeing. Hypotheses about the psychological benefits of horses (30) have been published, as well as the effects on cognitive and psychological factors (31), and post-traumatic stress symptoms (32, 33). Finally, EFT treatment for anxiety disorders has been used for adults (32, 34) and youth (35).

Positive relationships between the service provider /therapist and horse, and systematic training of horses for working situations are crucial for successful equine-facilitated services. In addition to impacting the horse's welfare, these interactions based on positive contact seeking with humans can affect the safety of work and the horse's contact-seeking with humans (36). For the horse to become a reliable and safe companion curiously seeking contact with the patient in the rehabilitation process the equine training needs to be based on continuous positive interactions and applying equine learning theory (37, 38). Negative affective states, inability to understand equine behavior and equine suboptimal living conditions not only play a great role in horse-related injuries and fear but can also hinder the horse's willingness to make contact with people (39).

The aim of this study is to evaluate the effect of a 12-weeks Equine-Facilitated Therapy intervention on chronic LBP patients' self-assessed functional impairments, wellbeing and pain. The hypothesis is that a 12-weeks intervention of EFT can relieve the amount of perceived pain and increase wellbeing and functional abilities of a chronic low back pain patient.

## 2. Materials and methods

The mixed methods design of the study was kept as close as possible to everyday clinical practice for LBP patients in EFT to ensure the replicability of the study. Quantitative and qualitative data were collected from the participants, who serve as their own controls. Measurements were taken before, during and after 12-weeks intervention. Final data collection, consisting of participant interviews and hospital data repository documentation, was completed 6 months after the treatment.

### 2.1. Participants

Twenty-seven subjects (4 male, 23 female), with a long history of clinical treatments for pain and decreased ability of daily functioning and working, were recruited by collaborating public hospital units. Participants that received a payment plan were pre-screened for any condition that might affect attending the intervention. The inclusion criteria were chronic low back pain within diagnosis groups ICD-10; M51.1-M54.5, and the exclusion criteria were acute spinal cord inflammation. One participant with primary diagnosis of multiple sclerosis was not included in the study, though did receive rehabilitation according to the payment plan. One participant did not show up and two participants withdrew during the intervention. The participants were informed of the study and signed a consent form. The study has ethical approval to perform a multiple center study (Dnro658/130100/16) from the Northern Ostrobothnia Hospital district and complies with the declaration of Helsinki.

### 2.2. Horse

Even though the horses already had experience working with humans and being ridden, a preparation to ensure the safety of the participants and the welfare of the horses was completed over 2014–2015. The horses were trained by applying learning theory into practice (40) (i.e., by gradually habituating to the equipment, humans and study place): shaping the responses to leading aids through negative (pressure and release) and positive reinforcement (e.g., food and scratching). The horses involved in the intervention were selected (41) according to the type of work they perform with the service provider. For this study, they were trained to work in a physical therapy setting, for example, to being accustomed to the patient's muscular imbalance. Additionally, the horses were accustomed to people entering and exiting the arena by opening revolving doors, filming and another horse (both familiar with each other) moving in different directions or staying in one place in the indoor arena.

### 2.3. Intervention

The intervention and outcome measurements were completed in two EFT centers in 2016–2017. Phone interviews were conducted on 22 (4 male and 18 female) participants and hospital patient data collected on visits to the rehabilitation or pain clinic.

The intervention consisted of 12 weekly EFT sessions. Given a delicate consideration of pain and its prolonged state, the exercise load (sitting on a moving horse) was increased systematically from 10 to 30 min: The first four times consisted of 10 min, next four times 20 min and last four times 30 min. During the first session, the patients were allowed to choose between two horses and equipment, both of which were pre-selected by the session leading therapist as the most suitable for them. The participants came to each allocated therapy session in pairs to encourage the social support of a “group”. The intervention was led by two physical therapists with a certificate in EFT and a long history in rehabilitation work. The indoor arena session consisted of two units: two participants, two therapists, two horses and two therapy assistants. Direct communication stayed within each unit, though all parties were aware of the other unit working in the same area. Although the session consisted of two units, they both worked independently.

**Figure 1:**
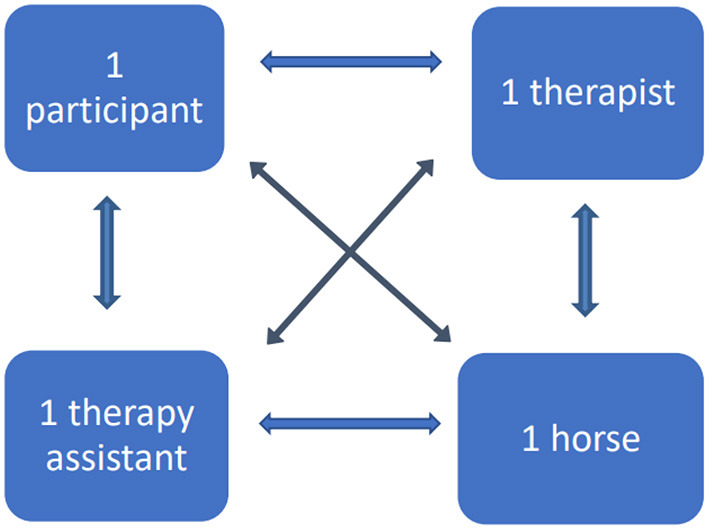
Single unit dialogue directions: The verbal and non-verbal communication lines. In the figure horses uses only non-verbal communications whereas all other are able to use both verbal and non-verbal communication simultaneously.

Every participant had five humans and two horses to be attentive to and each horse had six humans and another horse to observe. Each unit had 12 lateral, horizontal or diagonal verbal and non-verbal communication lines, as shown in Figure 1. The communication from the horses toward humans was non-verbal. Psychoeducation was completed in the form of multimodal feedback. The therapist used visual, vocal, kinesthetic and tactile feedback to interpret her analysis of the patients embodied understanding of the movement dialogue between the horse and the rider (42). The therapists filmed participants at the beginning and end of the intervention and interpreted from the together viewed video their walk and sitting posture on the horse. The indoor arena also had mirrors at the end and sides of the arena to view one's own posture during the exercise.

### 2.4. Data collection

The perspective of a patient is a core component of evidence-based practice (43). Questionnaires were completed pre- and post-intervention, an interview as well as the patient data repository visit were compiled at the end and a numeric rating of the amount of pain was collected twice every session to measure the possible effect.

#### 2.4.1. Questionnaires

The participants answered the questionnaires at home before they came to the first session of the intervention and after the last one. Questionnaires containing prefilled envelope and stamp with return address for the researcher were sent to the participants by mail. Performance rating with the help of an occupational therapist was completed on site at the stables.

Canadian Occupational Performance Measure (COPM) (44) helped participants to recognize and rate self-selected items (2–5) of daily functioning and satisfaction with functioning with numbers from zero to ten (0–10) (items that the participant wanted to do, those they needed, or were expected to do but could not do, and those they did not do or were not happy with how they did them). The bigger the number given, the higher the participants rated their functioning and satisfaction with functioning.

The quality of life - questionnaire (SF-36) was self-administered by the participants. The 36 -item pre-coded survey assesses the following eight health concepts: (1) limitations in physical activities because of health problems; (2) limitations in social activities because of physical or emotional problems; (3) limitations in usual role activities because of physical health problems; (4) bodily pain; (5) general mental health (psychological distress and wellbeing); (6) limitations in usual role activities because of emotional problems; (7) vitality (energy and fatigue); and (8) general health perceptions (45). SF-36 results are significant when at least one of the subdomain scores reports a statistically significant result in favor of the experimental intervention (46).

Similarly, the short version of Beck's depression questionnaire (R-BDI) (47) is a self-reported test consisting of 21 items on physical, behavioral and cognitive symptoms of depression from mild to severe (0–63 scores). The form is internationally validated to indicate depression. The questionnaire contains one question of anxiety that is not used to calculate the depression index. Scores between 0 and 13 indicate no or minimal depression, 14 – 19 mild depression, 20 – 28 moderate depression and 29 – 63 severe depression (48).

Chronic Pain Acceptance Questionnaire (CPAQ) (49) evaluated self-reported chronic pain acceptance with two subscales and is valid and reliable to detect rehabilitation outcomes. The survey reflects on “Activity Engagement”, or the pursuit of life despite pain (scale 0 – 66) and “Pain Willingness”, a lack of effort to prevent or control pain (scale 0 – 54) (48).

#### 2.4.2. Unidimensional measure

The numeric rating scale (NRS) was used to verbally indicate the amount of pain experienced by each participant at the arrival to each session and shortly after descending from the horse. On the 11-point numeric scale, 0 represents “no pain” and 10 “worst pain imaginable” (50).

#### 2.4.3. Interviews

A semi-structured follow-up interview was implemented six months after the intervention to supplement the patient data repositories and record patient experiences of the intervention (51). The follow-up interview was voluntary and conducted by the researcher who was not steering the practical sessions of the intervention to remain neutral. Answers were collected on 1 day and the phone call lasted between two and 15 min depending on the individual's verbal expression. The interviews were written records of a completed oral discussion. The questions included, “*How have you been since the intervention?”; “Have you visited the pain clinic since the intervention?” and “What would you like to say about the intervention?”*.

#### 2.4.4. Patient data repository

Access to the hospital patient data repository was granted to the sending hospitals with a contract. The researcher secured an identity card with limited access to rehabilitation and pain clinic patient data repositories. The search was limited to the relevant ICD-10 diagnosis or pain symptom in the lumbar area that prompted the participant to join the intervention. The findings were documented at the hospital archives room in the presence of an archivist. A code of visit for each patient data was reported as “for the research intentions” with a date and a timestamp. Patient data repositories were visited within a week of the interview.

### 2.5. Statistical analysis

Paired *t*-tests were estimated using IBM SPSS 27.0 (IBM, Armonk, USA) to analyze the difference between the beginning and end measurements of the intervention and to show the outcome results of the rehabilitation. *P*-values < 0.05 were considered significant. The effect size was measured with Cohen's *d* also reporting the lower and upper confidence interval (52). A value of 0.2 represents a small effect size, 0.5 a medium effect size and 0.8 and above a large effect size. Pearson's correlation and Kendall's tau was performed on COPMs. A value >0 indicate a positive association between two variables.

### 2.6. Qualitative analysis

The analysis of interviews is qualitative and the content of the interview was independently reviewed by the first and last author of this article. The themes were analyzed and thematized into groups. Two sets of analysis were united into one convergent result highlighting the main themes and conditions that generated these themes at the individual and group level (53). Visits to the rehabilitation or pain clinic were counted of 22 participants patient data repository entries and compared to the data received from interviews.

#### 2.6.1. Final analysis

The final analysis of the results was completed by uniting qualitative and quantitative data together to validate different sources of collected information.

## 3. Results

The results are listed below according to the method used to detect the outcomes of the intervention. These first lists (4.1–4.5) the results of measured and statistically analyzed questionnaires and second reports the (4.6–4.7) analyzed data.

### 3.1. COPM

Canadian Occupational Performance Measure was used to measure those performances (d1) of daily life the participants wanted or needed to do but couldn't to their satisfaction (d2). The 22 participants selected altogether 106 important performances (2 - 5 self-selected items/participant). The most identified (20 times) within the group of participants were “sleep and sleep-related items”, like falling asleep and continuous sleeping without waking up (*p* = 0.037, d1 = 0.96 and d2 = 0.55). Tasks that require “reaching and bending forward” (d1 = 1.92) like vacuuming and lifting objects from the floor were identified second most (18 times). “Sitting for a long time”, for example in the car or at work, was identified 15 times, and satisfaction with sitting related functions was found significant (*p* = 0.037, d1 = 2.13 and d2 = 2.02). “Standing for a long period of time” in various everyday work and home situations was identified 10 times, and satisfaction with standing related functions was found significant (*p* = 0.039, d1 = 0.58 and d2 = 0.54). The rest of the identified items were selected under 10 times and were: performances needing “walking” (walking with a dog or to a shop) (d21 = 0.75); “Carrying, lifting and moving heavy objects needing power/strength”, for example carrying a bag from a shop and shoveling snow or working in the garden; “other exercise” like walking, running or skiing; “participation in free time activities” like hunting, dancing, concerts and reading; “taking part in social events” like hiking and meeting relatives, and “starting movement having been static” like sitting or lying down. The results are presented at Table 1.

**Table 1:** Canadian Occupational Performance Measure (COPM) *n* = 22.

**Domains of performance selected**	***p*-value**	**Effect size, Cohen's *d***	**Confidence interval**	
			**Lower**	**Upper**
Sleeping related functions (*n =* 20)	0.237	0.96	−0.22	0.90
Satisfaction with sleeping rel. func.	0.037	0.5	0.04	1.24
Bowing and reaching functions	0.058	1.92	−0.02	1.11
Satisfaction with bow and reac. function	0.119	0.45	−0.11	0.99
Sitting related functions	0.062	2.13	−0.30	1.20
Satisfaction with sitt. rel. func.	0.037	2.02	0.04	1.31
Standing related functions	0.373	0.58	−0.92	0.35
Satisfaction with stand. rel. func.	0.039	0.54	0.04	1.46
Walking related functions	0.838	0.28	−0.56	0.69
Satisfaction with walk rel. func.	0.138	1.75	−0.16	1.12

Significant at the 0.05 level. Effect size value 0.2 represents a small effect size, 0.5 a medium effect size and 0.8 and larger a large effect size.

The findings indicate that a positive change in self-selected items of performance and satisfaction with performance have a strong correlation (Pearson, *r* = 0.900, *p* < 0.001). The COPM measurements was tested again with Kendall's Tau τb. A strong association (τb = 0.677, *p* < 0.001) between COPM performance and satisfaction with performance was detected with Kendall's Tau.

### 3.2. SF-36

From the 36-item health survey, an increase was detected at a group level in general health (from 44 to 48%), physical functioning (from 55 to 57%), physical role functioning (from 39 to 52%), emotional role functioning (from 56 to 65%), vitality (from 48 to 51%), social functioning (from 66 to 71%) and bodily pain (from 39 to 41%). Mental health scores (MCS) indicated significant increase in mental health (*p* = 0.001) (Table 2).

**Table 2:** Quality of life questionnaire SF-36, *n* = 22.

**Domains of questionnaires**	***p*-value**	**Effect size, Cohen's *d***	**Confidence interval**	
			**Lower**	**Upper**
Physical Change Scores (PCS)	0.131	0.23	−0.10	0.76
Mental Change Scores (MCS)	**0.001**	0.36	0.30	1.26

Significant at the 0.05 level, marked bold. Effect size value 0.2 represents a small effect size, 0.5 a medium effect size and 0.8 and larger a large effect size.

### 3.3. RBDI

In the beginning, eight participants indicated no or minimal depression; this number increased to eleven (from 38 to 53%) by the end. The number of participants with mild depression changes were noted from six to four participants (from 29 to 19%), moderate depression changed from five to four (from 24 to 19%) and the number of severe depression participants stayed at two (9%). The sum of group-level points decreased from 150/1323 to 121/1323. Anxiety in the group level decreased from 15/63 to 12/63. The total scores detected significant change (*p* = 0.014) (Table 3).

**Table 3:** RBDI: Short version of Beck's depression inventory by Raitasalo*, n* = 22.

**Domains of questionnaires**	***p*-value**	**Effect size, Cohen's d**	**Confidence interval**	
			**Lower**	**Upper**
Depression	0.056	0.27	−0.87	0.01
Anxiety	0.329	0.16	−0.63	0.21
Total Score	**0.014**	0.22	−1.02	−0.11

Significant at the 0.05 level, marked bold. Effect size value 0.2 represents a small effect size, 0.5 a medium effect size and 0.8 and larger a large effect size.

### 3.4. CPAQ

Physical functioning increased in the group level from 54 to 58 points, however the mental functioning indicated pain acceptance decreased from 40 to 35. The total score for chronic pain acceptance increased from 46 to 48. The chronic pain acceptance questionnaire showed no statistical significance (Table 4).

**Table 4:** Chronic pain acceptance questionnaire, CPAQ, *n* = 22.

**Domains of questionnaires**	***p*-value**	**Effect size, Cohen's d**	**Confidence interval**	
			**Lower**	**Upper**
Physical functioning	0.07	0.16	−0.40	0.81
Mental functioning	0.738	0.06	−0.35	0.49
Total Score	0.113	0.13	−0.08	0.78

Significant at the 0.05 level. Effect size value 0.2 represents a small effect size, 0.5 a medium effect size and 0.8 and larger a large effect size.

### 3.5. NRS

**Figure 2:**
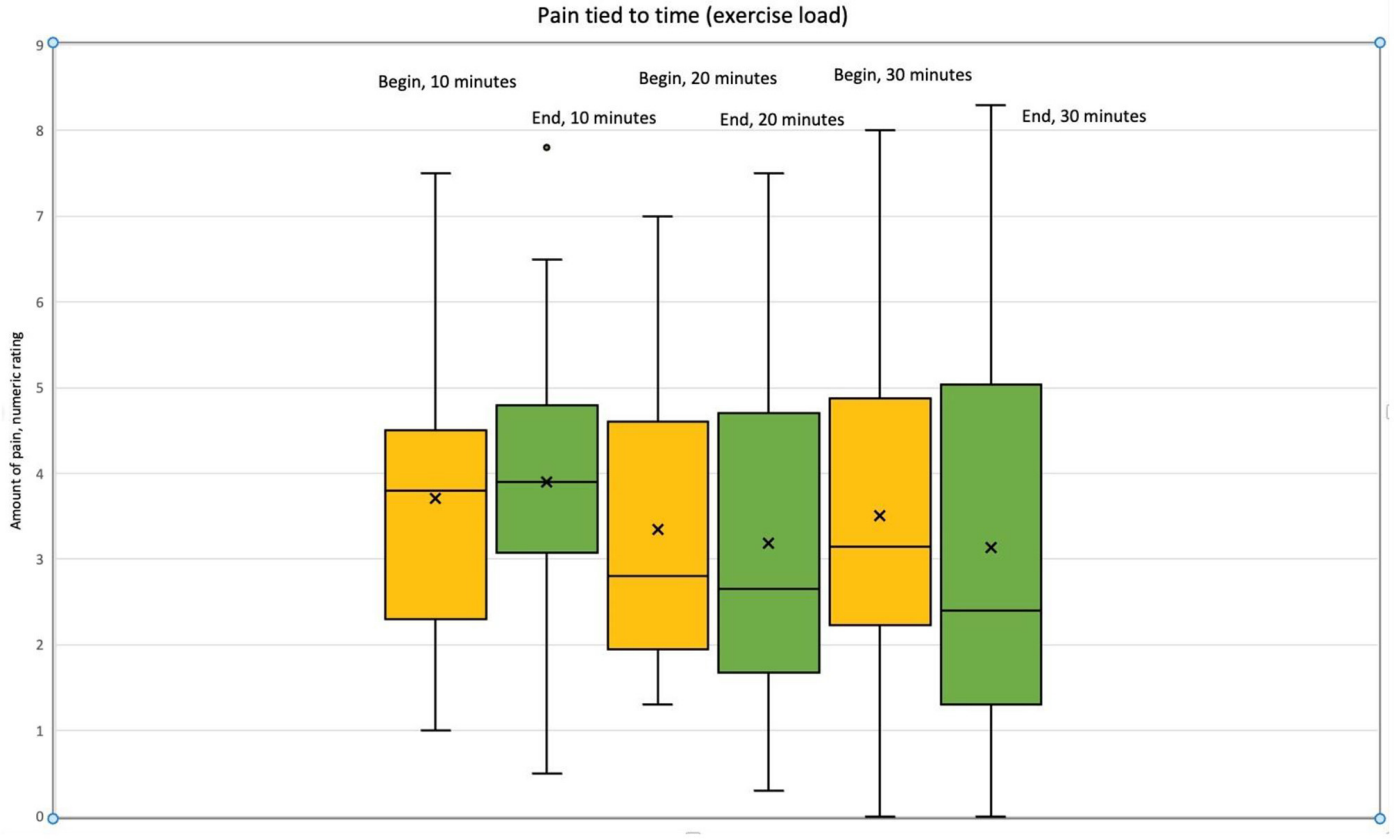
Exercise vs. pain chart.

A numeric rating scale for the amount of pain was analyzed considering the exercise length of time (from 10 to 30 min). The amount of pain decreased with the increase of the load and progression of the intervention. The decrease in the amount of pain during the intervention was found significant (*p* = 0.050, *d* = 0.74). Exercise response was found most significant following 30 min on horseback (Table 5 and Figure 2).

**Table 5:** Numeric rating scale (NRS) exercise response vs. amount of perceived pain, *n* = 22.

**Exercise Time on Horseback**	***p*-value**	**Effect size, Cohen's d**	**Confidence interval**	
			**Lower**	**Upper**
10 min	0.712	0.08	−0.34	0.50
20 min	0.381	0.08	−0.61	0.23
30 min	**0.032**	0.74	−0.93	−0.04
Total	0.050	0.63	−0.67	0.18

Significant at the 0.05 level. Effect size value 0.2 represents a small effect size, 0.5 a medium effect size and 0.8 and larger a large effect size.

### 3.6. Interviews

**Figure 3:**
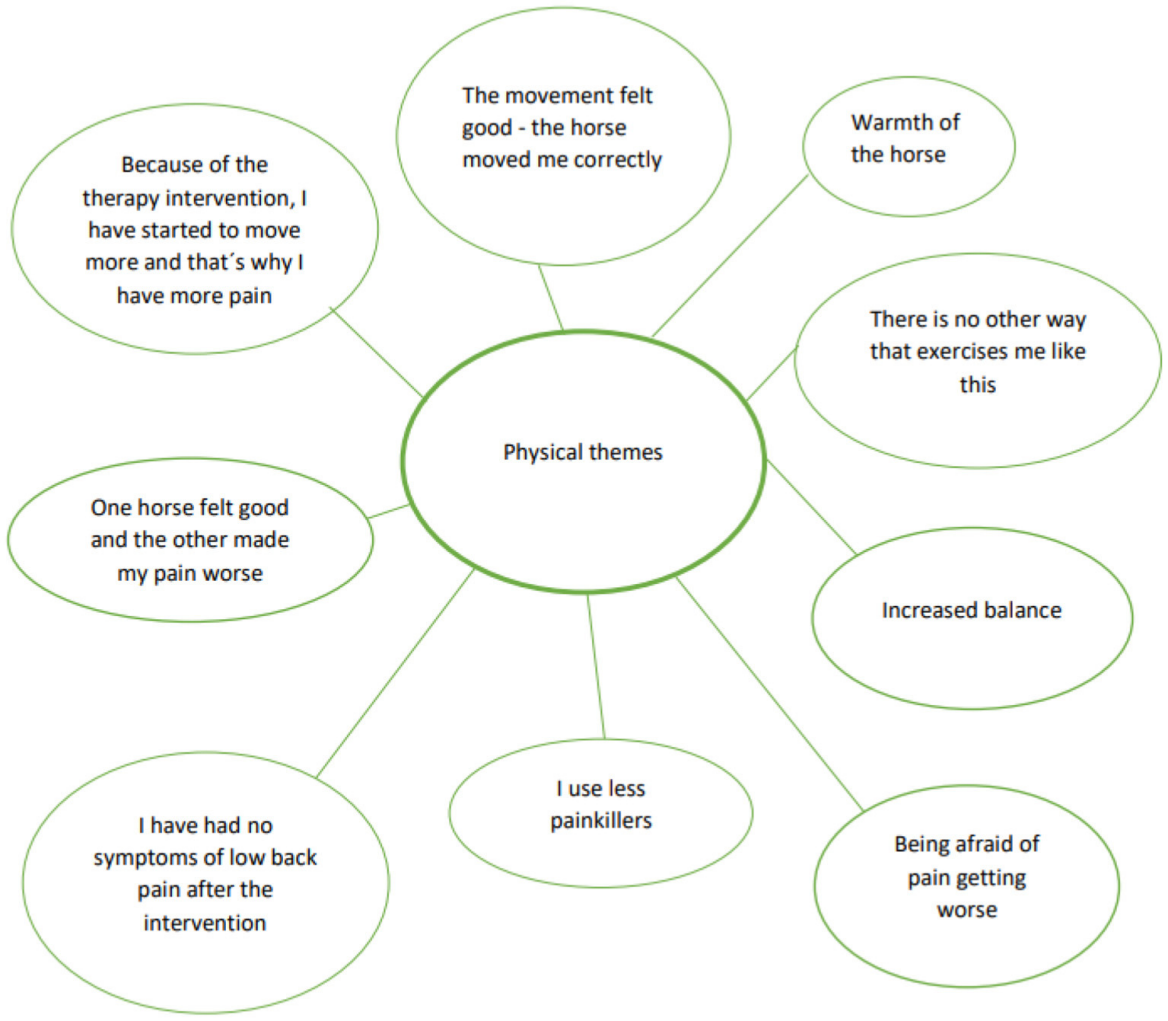
Physical themes.

**Figure 4:**
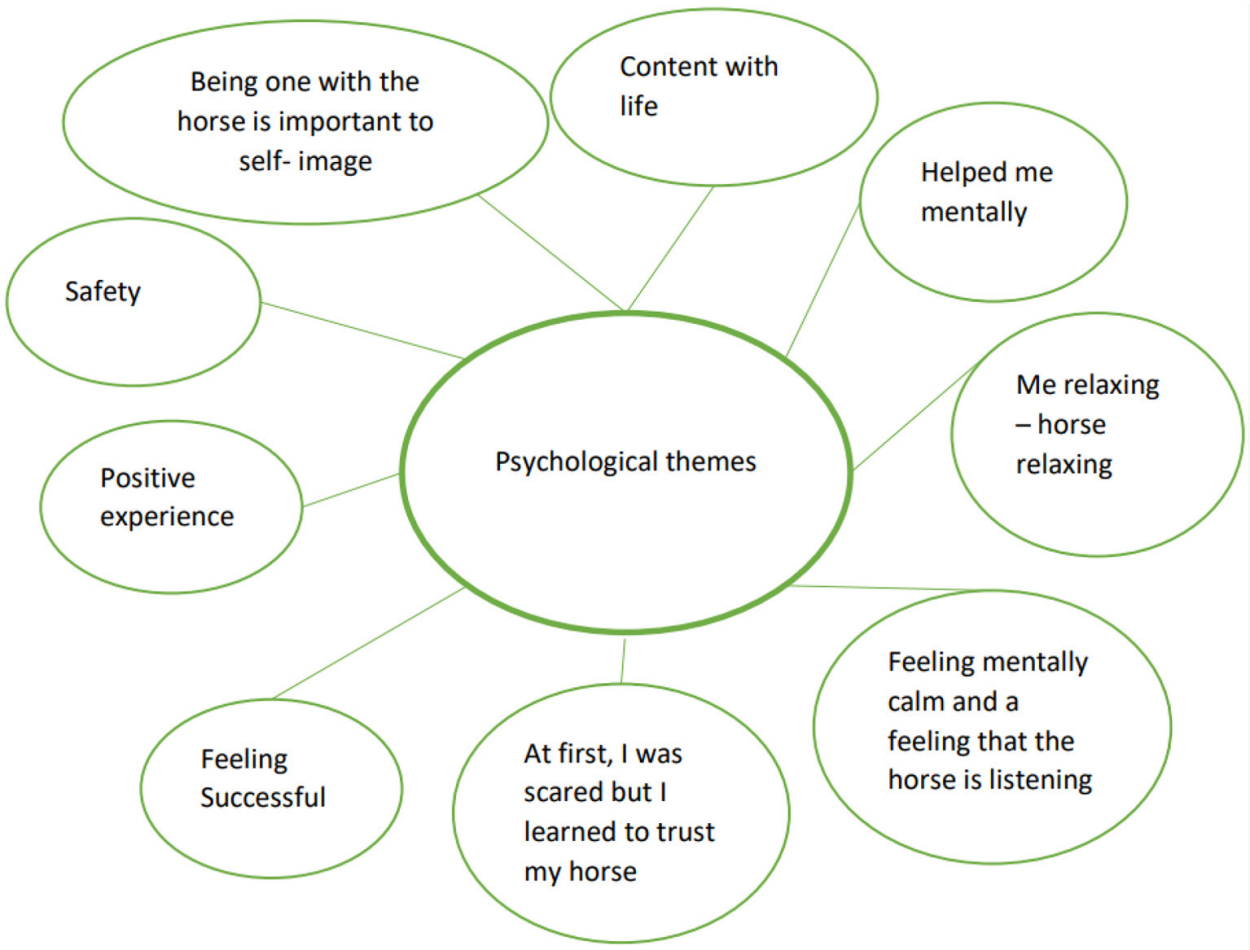
Psychological themes.

**Figure 5:**
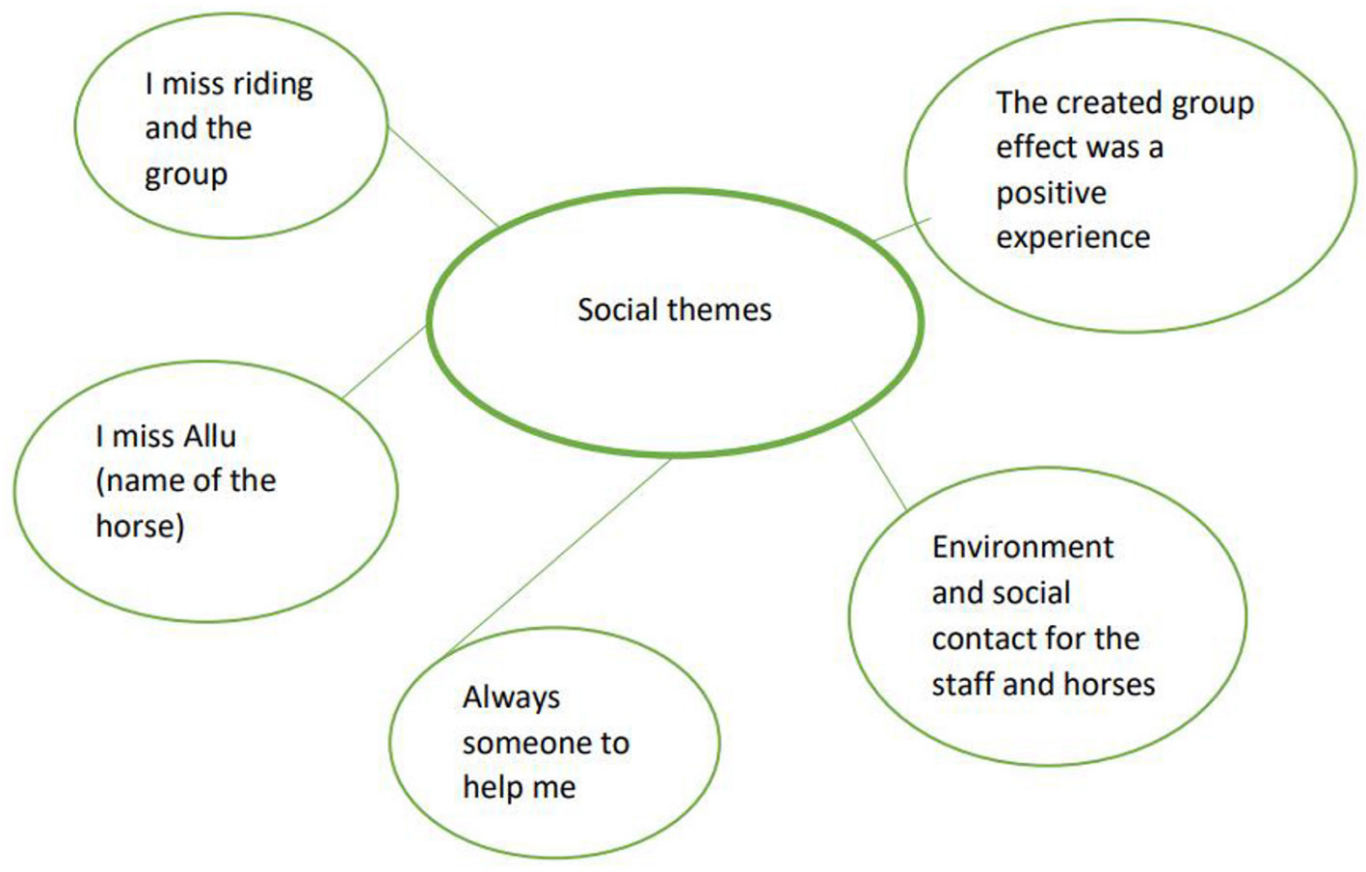
Social themes.

The participants answers revealed that they experienced a decrease in pain during the intervention with elevated levels of performance. The support of the units during the sessions had built a safe space and trust for rehabilitation. They also felt more self- confident adding daily physical performance and grew more satisfied with performance. The participants were not diagnosed with mental health problems, but answers reveal that the intervention helped their mental health. Three main themes were detected in line with research question: physical (Figure 3), psychological (Figure 4) and social themes (Figure 5).

### 3.7. The patient data repository

Reviewing the patient data repositories revealed that only two of the 22 participants had visited the health care center, with the same problem for which they were sent to the EFT intervention for during the 6 months after the intervention had ended.

## 4. Discussion

The aim of this study was to evaluate the effect of 12 weeks Equine-Facilitated Therapy intervention for chronic LBP patients' self-assessed functional impairments, wellbeing and pain. The participants served as their own controls in the 12 weeks intervention. The material was collected pre- and post- intervention added with a control in the form of interviews and archive search 6 months after the intervention. The results indicate the primary outcome was a strong correlation between performance and satisfaction with performance. Secondarily, the mental health scores and quality of life improved. Thirdly, the total score of depression inventory decreased. In the study completed by Ojala et al. (54) participants indicated pain to be “the phenomenon that affects the whole person”. Health-related follow-up information from the interviews, which came directly from the patient, were found to contain themes that the participants had started to process during the intervention. The comments of patient interview are written in italic and tied to the measured results and previous research in this field.

### 4.1. Physical themes

#### 4.1.1. Amount of pain

The International Association for the study of pain has revised the definition of pain to be multifactorial: biological, psychological and social factors that contribute to the pain syndrome (55). In this study, the amount of pain was reported twice during every session (24 times), and pain acceptance using the CPAQ and bodily pain using the SF-36 were collected before and after the intervention.

The follow-up interviews revealed a long-lasting effect on the patients; “*I have had no symptoms of low back pain after the intervention”*. The patient data repositories complimented this finding: only two of the 22 had returned to the rehabilitation or pain clinic after the intervention. From the socioeconomical and individual level, managing life and pain (7) is important, and the findings of the study corroborate this: “*Because of the therapy intervention, I have started to move more, and that's why I have more pain”*.

A change in the finding of prolonged use of generic medication (6); “*I use less painkillers”* is individual, although the group level finding suggests that the increase in exercise load from 10 to 30 min was decreasing the amount of perceived pain and intake of medication. Even if the optimal time was found to be 30 min, the exercise load for the LBP patients must grow gradually. The turning point for average pain intensity seemed to be 20 min on the horseback and 5–8 weeks of intervention. This needs further research.

#### 4.1.2. Physical performance

External stimulus, like moving a body part that is painful, is generally avoided (4). Although, sitting on a moving horse and receiving 100-120 walk-like movements (21) was endorsed: “The movement felt good - the horse moved me correctly” and “There is no other way that exercises me like this”. The participants accepted one horse's walking phases correlating to own body movement (56) better than the other; “One horse felt good and other made my pain worse.” The ability to connect professional's and patient's expertise in the selection of the horse and equipment seems to be important in pain - related rehabilitation. The “Warmth of the horse” has been found to be 1.5 degrees higher than in humans and affects muscular tone (57). It may have influenced a COPM measured stationary performance, Sleeping related functions, selected considerably frequently 20/22 amongst the participants. Sleeping quality is not an indicator for decreased amount of pain, however having an association between sleep quality, low back pain and disability (58) and an association between sleeping problems and the intensity of chronic pain (59) has already been found.

A movement dialogue between the patient and the horse is the primary interest in EFT from physical therapy perspective. Psychoeducation for the participants through video gave visual feedback of bodily movements (60)in walk and sitting on the horseback. Especially the movement that the horse produced for the LBP patient; “*I didn't know my back could move that much*” seemed to be important psychoeducation to overcome the fear of movement.

EFT is reported to increase postural balance and to improve everyday performance (61). “*Increased balance”* was supported at the patient interview with the increase of COPM daily performances: bowing and reaching, sitting, standing and walking. Kinesiophobia, irrational fear of movement motivates defensive and avoiding behavior toward exercise and everyday performances with elevated levels of pain (3);“*I have been afraid of pain getting worse”*. The results suggest small increase to physical functioning according to SF-36 Physical Change Scores and CPAQ. An interest toward interventions is increasing where one's awareness, acceptance, internal experiences, and physical sensations are enhanced (12).

### 4.2. Psychological themes

People with elevated pain-related anxiety and fear avoid activities that may be important to recovering (1). “*Feeling successful”* reveal the developing emotional self-competency. Emerged sentences; “*Content with life”* and: ”*Positive experience”* might be indicators of emotional awareness and comply with Lumley et al. (12) research of emotion producing (12) and are supported by elevated RBDI depression and SF-36 Mental Change Scores indicating better mental health. Nevertheless, RBDI anxiety and CPAQ mental functioning address the opposite. The satisfaction with performance measured with COPM was evidenced more often significant than the performance tied to the satisfaction.

Positive emotional states generally reduce pain (62). The EFT intervention seems to have started emotional processes and created “*Safety”*(17) and “*Helped me mentally”* for the recovery. The interview reveals that the presence of the horse in the rehabilitation made the difference (63) for bonding “*At first I was scared but I learned to trust my horse”*, “*Mentally calm feeling and a feeling that the horse is listening”; “Being one with a horse is important to self-image”* (64) “*Me relaxing- horse relaxing”* (64) that are connected to the descriptions of human-animal attachment theories.

### 4.3. Social themes

With the horse and in the context of EFT as a form of physical therapy, assistant is utilized to provide best practice for the rehabilitation. This setting formed a unit of four (participant, horse, therapy assistant and therapist) living creatures where communication, verbal and non-verbal, was one of the key elements in the patient's experience of rehabilitation. As two same working units were present in the indoor arena; “*The created group effect was a positive experience”* and “*I miss riding and the group”*. The communication flew into 12 different directions allowing:“*Environment and social contact for the staff and horses”*. As the pain affects social interaction (2) and the patients feel isolated from normal daily activities and helpless because of the lack of physical performance;“*Always someone to help me”* supported the rehabilitation. These interview findings are supported by the results of SF-36 “limitations in social activities because of physical or emotional problems”. The intervention also added knowledge to the participants of themselves and their bodily movements, giving them confidence to perform physical activities and be satisfied with them enough to take part in social activities.

During the intervention the patients got to know the horses they had chosen as their therapy horses. A bonding and trust between the participant and the horse in therapy (65, 66) and “*I miss Allu (name of the horse)”* was recognized through the participant interveiw. Vice versa, as the horses became accustomed to the work, trusted the therapy assistant and the therapist, working was made predictable and safe, building a collective trust within the session (67).

### 4.4. The horse

We wanted to express the timely discussion of equine welfare in Equine - Assisted Interventions. If the welfare of the horse is not considered at the rehabilitation process, the justification of the use of the horse is not possible. Through careful selection of the horse (41) to be trained, and a proper training (40), EFT as a rehabilitation method can be beneficial reciprocally (68). The movement of the horse (21) added with the contact (36) between the patient and a horse, as well as the social support of the units during the sessions was found important for the participants during their recovery process. Some results, obtained with solely having the living creature, horse, present at the rehabilitation process might have been unattainable using electronic device as a substitute. The nature, natural bonding and equine environment have been found advantageous during the procedure (69).

As the field of Equine - Facilitated Interventions (EFI) has been growing rapidly not only terminology (70) but also variability in the levels, content and access to education bring complexity for service buyers, customers, service providers and horses affecting the brand. Recently ended EU-project: “Equine - Facilitated Interventions - Education” brought together five partners sharing the key elements of their education curricula which formulated the base of the results of the project leading to uniformity in EFI education. Another EU-project concentrating in equine training for different EFI disciplines will be completed with results of training consensus.

### 4.5. Strengths and weaknesses of the study

The strength of this study is that two of the authors work providing EFT, and one has been training therapy horses. Using several variables, both objective quantitative and subjective qualitative methods enable versatile interpretation of the 12 weeks intervention outcomes. The recruitment through public health care made the participants equal from medical and economical view. All collected and analyzed data point to the same direction of outcome and is connected to formulate a broader understanding of the rehabilitating effect of the horse.

Even though CPAQ did not detect acceptance to pain on the group level, it revealed differences in individual level, seen in the range of confidence interval. The COPM measurements helped the participants to identify the performances and satisfaction with performances of daily living that correlated in the statistical analysis. As the occupational therapist that had training for the COPM measurement, we believe objectivity within the data collection.

The study has limitations which should be considered when interpreting the results. Small sample size and lack of a control group are weaknesses. Other instruments could have been more relevant to use and midterm measurements could have provided us with more data. Repeated questions of pain twice every session may have led to unintended focus on pain and contradictory to the participants acceptance. Deeper interviews would have provided more qualitative data. This will be considered in future studies.

An important factor in considering strengths and weaknesses in this study is that the researcher assumed the intervention to produce mainly physical outcomes for the chronic low back pain patients. However, the byproduct caused by the interview 6 months after the intervention turned out to be core element of the study. The importance of human-animal interaction with meaningful experiences in physical, psychological and social domains became visible in the interview, shown in Figures 3–5.

In the future, a study where multi-professional team: Equine Facilitated therapists with mental and physical competence, with the horse and assistant is recommended. A longer intervention, repeated re-evaluated measures, psychoeducation and better prepared interview with control group receiving standard care, would produce more information for EFT as a rehabilitation and support recovery of the LBP patients.

## 5. Conclusions

The objective of this article is to evaluate the impact of EFT on perceived physical performance, pain and psychological wellbeing for 22 chronic LBP patients. Given the prolonged nature of the pain in the patient group the rehabilitation process of 12-weeks is considerably short. However, this study presents a mixed methods study where triangulation of data demonstrates how results from many sources, with only few significant changes give evidence for a successful treatment modality. The unique contributions from horses are presented from the data obtained.

The communication flow within the unit in the sessions, both verbal and non-verbal encouraged learning in performing physical daily activities, understanding bodily movement and self – confidence in the chronic LBP patients. Additionally, these participants have added knowledge to their ability to take part in social activities.

“*I would recommend it to other low back pain patients*” highlights a feeling of a participant for nature based active rehabilitation (69). Our suggestion is to continue with these chronic pain patients' themes, which would include psychotherapeutic exercises and elements that target pain management, mindfulness and self-compassion together with movement training.

## Data availability statement

The raw data supporting the conclusions of this article will be made available by the authors, without undue reservation.

## Ethics statement

The studies involving human participants were reviewed and approved by the Northern Ostrobothnia Hospital district. The patients/participants provided their written informed consent to participate in this study.

## Author contributions

All authors listed have made a substantial, direct, and intellectual contribution to the work and approved it for publication.
